# Adolescent-parent communication on sexual and reproductive health issues and associated factors among high school students in Woldia town, Northeastern Ethiopia

**DOI:** 10.11604/pamj.2018.31.35.13801

**Published:** 2018-09-18

**Authors:** Molla Temere Mekonen, Hana Abebe Dagnew, Tesfay Ambaye Yimam, Hayat Nuradis Yimam, Melese Abate Reta

**Affiliations:** 1 Department of Nursing, Faculty of Health Science, Woldia University, Woldia, Ethiopia; 2 Department of Medical Laboratory Science, Faculty of Health Science, Woldia University, Woldia, Ethiopia

**Keywords:** Adolescent, parent, communication, SRH issues, associated factors, Woldia town

## Abstract

**Introduction:**

Sexual and reproductive health (SRH) communication is most likely to promote healthy sexual practices and to reduce risky sexual behavior among adolescents. Communication is the principal means for parents to transmit sexual values and knowledge to their children. This study aimed to assess adolescent-parent communication on SRH issues among high school students in Woldia town.

**Methods:**

A cross-sectional study was conducted from December 15-31, 2016. Systematic random sampling technique was used to select 693 students from Grades 9 and 10. Data were entered into Epi-info version 3.5.1 and analyzed by using SPSS version 20. Logistic regression with Odds Ratios (OR) and 95% Confidence intervals (CI) was used to identify the independent predictors of adolescent-parent communication.

**Results:**

674 students accepted to participate in this study giving a response rate of 97.3%. Only 205(30.4%) had discussed on two or more SRH topics with their parents. In logistic regression analyses, mothers who could read and write [AOR=2.0; 95% CI=1.3-3.1] and had diploma certificate [AOR=2.0; 95% CI=1.4-2.9] were more likely to discuss on SRH issues with their children. Adolescents who agreed on the importance of discussion on SRH issues [AOR=2.5; 95% CI=1.3-4.5], whoever got SRH information [AOR=2.0; 95% CI=1.4-2.9] and those whoever had sexual intercourse [AOR=1.7; 95% CI=1.1-2.6] were more likely to discuss on different SRH issues with their parents.

**Conclusion:**

This study revealed that parent-adolescent communication on SRH issues was inadequate. Therefore, there is a need to equip and educate parents on different adolescents' SRH issues.

## Introduction

An increased incidence of sexually transmitted disease (STD) including human immunodeficiency virus (HIV) infections in adolescents in developing and developed countries has led researchers to examine factors that influence young people's sexual behaviors [1]. Improving parent-adolescent communication at all community level is important and it is a current agenda to all concerned bodies in all country to promote healthy sexual behaviors of adolescents [2]. The world health organization (WHO) report on the analysis of adolescents SRH literature from different parts of the world informs that this concern has been largely driven by the high prevalence of HIV/AIDS among young people [3]. Though, sexual and reproductive health communication between parent and adolescent is a most important way of conveying sexual values and knowledge [4], discussions on sexual and reproductive health issues particularly sex-related matters are unacceptable and shame in most African countries [5] and parents believed that advising adolescents about sex-related issues and updating them how to abstain would make the adolescents sexually active [2, 6]. Most youth-friendly services and health care providers in Ethiopia are not well prepared in addressing adolescents' sexual and reproductive health desires. Hence, parents' communication with their children about SRH issues is important to enhance adolescents' awareness and to reduce their risky sexual behaviors [6]. Parent-adolescent communication on SRH issues in Ethiopia is believed to be socially disgraceful; moreover, parents are not open and uncomfortable to discuss these issues with their young children [7]. Parents' low level of knowledge about sexual and reproductive health issues makes open discussions with their children challenging. Nevertheless, as different studies revealed that home is the preliminary place to educate adolescents about SRH issues, and it is the first social environment for them [5].

Different Studies revealed that adolescents in Ethiopia have very low health-seeking behavior mainly to their SRH matters and even the current reproductive health services are not adolescent-centered [6, 8]. Furthermore, health care workers in Ethiopia are not well prepared to address adolescents' SRH desires [8]. Hence, the involvement of families, community members, and other stakeholders is important to improve the health status of the adolescents [9]. Thus, families, as primary socializing agent and live models for their children need to play an important role in shaping the sexual life of their adolescents and to reduce their burden of diseases and disabilities associated with SRH [6, 10]. However, not much support is offered for parent-adolescent communication, and parents often do not discuss with their children because they feel confused, ill-informed, or embarrassed about SRH topics [11]. Even though the Ethiopian government has identified the reproductive health of adolescents as one of the priority areas in the national RH Strategy taking the households and community as vehicles for change, it is not yet put in practice [12]. Parental discussion about adolescents' SRH matters is very important perhaps now than any other time. This is because adolescents are affected by the burden of unwanted pregnancy and its complication, HIV/AIDS, sexual transmitted infections (STIs) and other sexual and reproductive ill health to a greater extent [6, 13]. Therefore, the aim of this study was to determine adolescent-parent communication level on SRH issues and to assess associated factors among high school students in Woldia town, Northeastern Ethiopia.

## Methods

**Study area and period:** The school-based cross-sectional study was conducted among high school students in Woldia town, North Wollo Zone, Amhara Regional State from December 15-31, 2016. Woldia town is located in the Northeastern part of Ethiopia at 521km from the capital city, Addis Ababa. According to 2007 Ethiopian population census report, the total population of the town was 46,139 with sex distribution of 49.8% “males” and 50.2% “females”. Moreover, from the total population, 13,027(28%) found in the age group of 15-24 for both sexes [14]. The town has two high schools namely Woldia high school and Millennium high school in which grade 9 and 10 education is delivered. Accordingly, Woldia high school and Millennium high school had a total of 1,844 and 1,195 students during the study period, respectively. There are one hospital and two health centers in the town, but there are no well-equipped youth friendly centers.

**Study population:** The study population was all students of grade 9 and 10 of both high schools in Woldia town during the study period. Unmarried adolescents in the age group 15-24 years were included in the study. The sick, visually impaired and those unwilling to participate were excluded from the study.

**Sample size determination:** The sample size was calculated using single population proportion formula considering the following assumptions: P=28.9% [13], significance level 5% (α=0.05), and Z^α^/2=1.96, margin of error 5% (d=0.05). Assuming 10% non-response rate and design effect 2, the final sample size was 693.

**Sampling procedures:** A multi-stage sampling technique was used. Simple random sampling was used to select the sections from each grade in the two schools. To select the study unit, students" roster was used as a sampling frame. To determine the number of students from each grade, proportional allocation to their size was used and in every step, a simple random sampling technique was used to select the grade and students from each section. Finally, the study subjects were selected using simple random sampling technique.

### Study variables

**Dependent variable:** Levels of communication on SRH issues.

**Independent (explanatory) variables:** Determinant factors for parent-adolescent communication like socio-demographic characteristics (gender, age, living arrangement, family size, family income, parents" education, cultural norms); behavioral factors (perceived openness of parents to discuss on SRH issues); sexual behavior(early sexual initiation, unprotected sex, multiple sexual partners, unwanted pregnancy) were assessed.

### Operational definition

**Communication on SRH issues:** Students who discussed at least two SRH issues (condom, STI/HIV/AIDS, abstinence, unwanted pregnancy, contraception and premarital sex) with their parents in the last 12 months.

**Parents:** Biological parents, step-parents or foster parents including elder siblings.

**Adolescents:** Segment of the population who are unmarried and fall to the age range of 15-24years.

**Data collection instruments:** Data were collected using pretested structured self-administered questionnaire. The questionnaire consisted of socio-demographic characteristics of parents and adolescents, behavioral factors of parents, and sexual behaviors of the adolescents. The questionnaire was prepared in English, and it was translated into Amharic language (local language) for appropriateness and easiness. The Amharic version was again translated back to English language to check the content validity of the original version. Translation of questionnaire was done by language experts in both cases.

**Data collectors:** Three individuals who had completed grade 12, preferably who has experience of data collection were selected. Confidentiality and privacy were given attention during training and the trainees participated during pre-testing of the questionnaire. The aim of the study was explained to the selected adolescents. Data collectors were supervised by two B.Sc degree holder nurses and problems faced during data collection were solved on time. The principal investigator had checked filled questionnaires and solution was given by discussing with the supervisors and data collectors. Finally, filled questionnaires were signed by supervisors after checking for its completeness.

**Data quality control:** Data collectors were trained for one day on the objectives of the study, sampling procedure, about the questionnaire and how to check the completeness of questionnaires. To ensure the quality of the data, all the filled questionnaires were checked for incompleteness and inconsistency. The investigator also discussed with the supervisors on how to supervise the data collectors and how to solve problems encountered during the data collection process. Data collectors were supervised by B.Sc degree holder nurses. Moreover, pre-test was conducted on 10% of all the total sample size from other high school to ensure its completeness and consistency in providing the information needed for the study.

**Data processing and analysis:** In this study, the term “parent-adolescent communication on SRH issues refers to parents opening a discussion on SRH issues or parental involvement in SRH discussion initiated by the adolescents or both”. It was measured with a “Yes” for those who had a parental discussion and a “No” option for those who had no parental discussion. Data were coded, edited, and entered using EPI Info version 3.5.1 and then transported to SPSS version 20 for statistical analysis. Descriptive statistical analysis was used to compute frequency and a percentage of independent and dependent variables. Binary logistic regression analysis was used to ascertain the association between explanatory variables and outcome. Variables with significant associations in the bivariate analysis were entered into a multivariate analysis to determine the independent associated factors of adolescent-parent communication on SRH issues. Variables with a P value<0.05 was considered as significant.

**Ethical consideration:** Ethical clearance was obtained from Woldia University; Faculty of Health Science institutional research ethics review committee. Permission was obtained from school administration and schools' parent-teachers union. Informed verbal consent/assent was obtained from every participant after explaining the purpose of the study in detail. Confidentiality of information was kept anonymously.

## Results

**Socio-demographic variables of respondents:** The response rate for this study was 674/693(97.3%). Of these, 345 (51.2%) were females. The median age of the respondents was 18.96. Four hundred eighty-nine (72.6%) were Orthodox Christians. Adolescents who were living with their fathers and mothers account 480(71.2%) followed by those who were living with their mothers 102(15.1%). Three hundred sixty-nine (54.7%) were grade nine students followed by grade ten students who account 305 (45.3%) (Table 1). Only, 158 (23.4%) of the participants knew their families' monthly income. Three hundred forty (50.4%) and 194 (28.8%) of adolescents' fathers and mothers were illiterate, respectively. On the other hand, 410(60.8%) of the adolescents' mothers were housewife. Five hundred four (74.8%) of the adolescents' fathers were government employees (Table 2).

**Table 1:** Socio-demographic characteristics of high school students in Woldia town, December 2016 (n=674)

Variables		N	%
Sex	Male	329	48.8
	Female	345	51.2
Age	15-19	410	60.8
	20-24	264	39.2
Grade level	Grade 9	369	54.7
	Grade 10	305	45.3
Religion	Orthodox	489	72.6
	Muslim	172	25.5
	Protestant	13	1.9
	Catholic	0	0
	Others	0	0
Living Arrangement	Father &Mother	480	71.2
	Only with Father	102	15.1
	Only with Mother	22	3.3
	Brothers/sisters	0	0
	Others**	70	10.4
	Alone	0	0

** Others indicate those who live with friends, relative or other than either of their parents

**Table 2:** Socio-demographic characteristics of parents’ of enrolled adolescent high school students in Woldia town, December 2016 (n=674)

Variable		N	%
Educational status of mother	Illiterate	340	50.4
	Read & write only	73	10.8
	Primary school	124	18.4
	Secondary school	8	1.2
	Diploma	102	15.1
	Degree & above	27	4.0
Educational status of father	Illiterate	194	28.8
	Read & write only	30	4.5
	Primary school	70	10.4
	Secondary school	135	20.0
	Diploma	75	11.1
	Degree & above	170	25.2
Mother’s occupation	Housewife	410	60.8
	Gov’t Employee	227	33.7
	Private employee	21	3.1
	Merchant	16	2.4
	Others	0	0
Father’s occupation	Unemployed	46	6.8
	Gov’t Employee	504	74.8
	Private employee	22	3.3
	Merchant	70	10.4
	Others	32	4.7
Family size	<5	156	23.1
	5 & above	518	76.9
Parents’ Monthly Income (Birr)	<1000	44	6.5
	1000-2500	54	8.0
	2501-3500	52	7.7
	3501-4500	8	1.2
	>4500	0	0
	Don’t know	516	76.6

**Figure 1:**
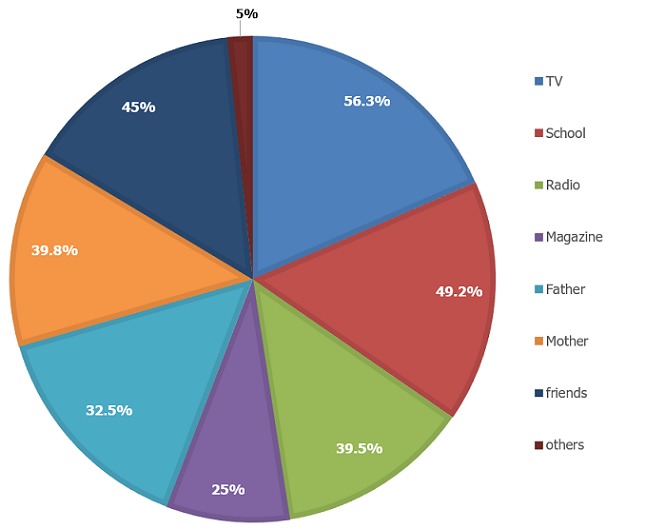
Source of SRH information for high schools’ students in Woldia town, December 2016 (n=674)

**Adolescent-parent communications on SRH issues:** Majority of the adolescents, 528(78.3%) believed that it is important to discuss on SRH matters with their parents. Parents are recommended to have regular communication on SRH issues with their adolescents. However, only 205(30.4%) of adolescents had a discussion with either of the parents on at least two topics of SRH issues (Table 3). The adolescents' estimated reason for not discussing contraceptive with their parents was culturally unacceptable 191(28.3%). The other reason that adolescents not discussing about contraceptive with their parents was feel ashamed 151 (22.4%) (Table 4). Majority of the adolescents, 447(66.3%) had no discussion about STIs/HIV/AIDS because Parents had less communication skill to discuss on SRH issues 199(29.5%) and lack of knowledge 132(19.6%) (Table 4). Only, 111(16.5%) of adolescents had discussed about sexual intercourse with their parents. Among those, 98(14.8%) had discussed with their friend/s. Eighty one (12%) adolescents reported that they had a discussion about unwanted pregnancy. Among those, 67(9.9%) had discussed unwanted pregnancy with their friends, and 62(9.2%) with their mothers. Two hundred fifty six (38.0%) of adolescents reasoned out that, they felt ashamed to discuss unwanted pregnancy with their parents (Table 4). Among 129(19.1%) of the adolescents who had a discussion on premarital sex, 121(18.0%) and 119(17.7%) had a discussion with their friends and mothers, respectively (Table 3). Five hundred forty five(80.9%) of the adolescents didn't discuss about premarital sex; among those, 122 (18.1%) of the adolescents didn't know the reasons why they didn't discuss and 188(27.9%)of the adolescents said it was shame to discuss about premarital sex with their parents (Table 4). On the other hand, 65(9.6%) of the adolescents claimed to have had a discussion on a condom. Fifty seven (8.5%) adolescents discussed with their friends, followed by their mothers 55(8.2%). Most of the adolescents had no discussion on a condom, the reasons they mentioned were feel ashamed 230(34.1%), and culturally unacceptable 109 (16.2%) (Table 4). Concerning the preferred groups for discussion about SRH issues, 327 (48.5%) of the adolescents chose their friend/s followed by mothers which account 324 (48.1%) (Figure 1). Two hundred nine (31.1%) reported that mothers were open to discuss SRH issues when compared to fathers 113 (16.8%). On the other hand, 311(46.1%) of adolescents rated their parents communication skill on SRH issues as low whereas, 136(20.2%) and 55(8.2%) students said medium and high, respectively. One hundred forty one (20.9%) adolescents had discussed with their parents on contraceptives. Among those, 135(20%) of them had a discussion with their peers followed by mothers 125(18.5%). The adolescents who had had a discussion on STIs/HIV/AIDS were 227(33.7%), and of those, 188(27.9%) had a discussion with their friends, followed by their mothers 140(20.8%) (Table 5).

**Table 3:** Different SRH issues discussed by adolescent high school students in Woldia town, December 2016 (n=674)

SRH issues discussed		Male(n(%))	Female ( n(%))	Total ( n(%))
Importance of discussion on SRH	Agree	265(80.5)	263(76.2)	528(78.3)
	Disagree	64(19.5)	82(23.8)	146(21.7)
Discussion on at least two SRH issues	Yes	108(32.8)	97(28.1)	205(30.4)
	No	221(67.2)	248(71.9)	469(69.6)
Sexual intercourse	Yes	59(17.9)	52(15.1)	111(16.5)
	No	270(82.1)	293(84.9)	563(83.5)
Unwanted pregnancy	Yes	43(13.1)	38(11.0)	81(12.0)
	No	286(86.9)	307(89.0)	593(88.0)
Contraceptive	Yes	76(23.1)	65(18.8)	141(20.9)
	No	253(76.9)	280(81.2)	533(79.1)
STI/HIV/AIDS	Yes	108(32.8)	119(34.5)	227(33.7)
	No	221(67.2)	226(65.5)	447(66.3)
Premarital sex	Yes	59(17.9)	70(20.3)	129(19.1)
	No	270(82.1)	275(79.7)	545(80.9)
Condom	Yes	43(13.1)	22(6.4)	65(9.6)
	No	286(86.9)	323(93.6)	609(90.4)

**Table 4:** Reasons for adolescent high school students for not discussing on SRH issues with their parents in Woldia town, December 2016 (n=674)

Topic of discussion	Reason for not discussing						
	Not discussed	*Culturally unacceptable	*Shame	*Parents have less comm. Skill	*Parents lack knowledge	*Fear of parents	*Parents aren’t good listener
Contraceptive ^ǂ^	533(79.1)	191(28.3)	151(22.4)	68(10.1)	112(16.6)	87(12.9)	92(13.6)
Sexual intercourse^ǂ^	563(83.5)	111( 16.5)	230(34.1 )	56(8.3 )	45(6.7 )	190( 28.2)	90( 13.4)
STI/HIV/AIDS^ǂ^	447(66.3)	69(10.2)	112 (16.6)	199 (29.5)	132(19.6)	45(6.7)	52(7.7)
Unwanted pregnancy^ǂ^	593(88.0)	127(18.8)	256(38.0)	93(13.8)	106(15.7)	60(8.9)	51(7.6)
premarital sex ^ǂ^	545(80.9)	104 (15.4)	188(27.9)	79(11.7)	83(12.3)	97(14.4)	64(9.5)
Condom^ǂ^	609(90.4)	109(16.2)	230(34.1)	99(14.7)	89(13.2)	93(13.8)	69(10.2)

* Multiple responses were possible

ǂ percents are in the bracket

**Table 5:** Persons with whom adolescent high school students discuss SRH issues in Woldia town, December 2016

Topic of discussion	Discussed	With whom they had discussed				
	Yes	Father*	Mother*	Friend*	Brother*	Sister*
Contraceptive^ǂ^	141(20.9)	45(6.7)	125(18.5)	135(20.0)	1(0.1)	34(5.0)
Sexual intercourse^ǂ^	111(16.5)	21(3.1 )	98(14.1 )	98(14.8)	15(2.2)	55(8.2 )
STI/HIV/AIDS ^ǂ^	227(33.7)	65(9.6)	140(20.8)	188(27.9)	78(11.6)	100(14.8)
Unwanted Pregnancy ^ǂ^	81(12.0)	34(5.0)	62(9.2)	67(9.9)	19(2.8)	39(5.8)
Premarital sex ^ǂ^	129(19.1)	45(6.7)	119(17.7)	121(18.0)	22(3.3)	53(7.9)
Condom ^ǂ^	65(9.6)	40(5.9)	55(8.2)	57(8.5)	28(4.2)	35(5.2)

* Multiple responses were possible

ǂ percents are in the bracket

**Figure 2:**
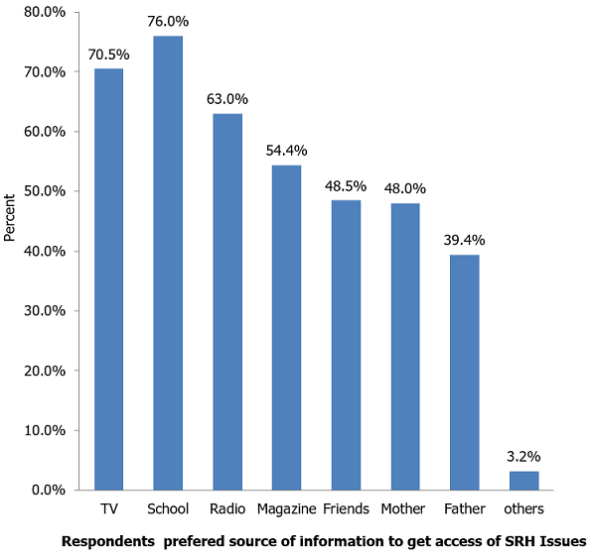
Adolescents’ preferred source of information to get access to SRH issues in Woldia town high schools, December 2016 (n=674)

**Source of information for SRH issues:** Four hundred forty six (66.1%) of adolescents mentioned that they got SRH information. Among mentioned source of information for SRH issues, Television, and school account 332 (56.3%) and 293 (49.2%), respectively (Figure 1). Only, 48.5% and 48% of the adolescents reported that friends and mothers were their SRH issues information sources, respectively. Majority of participants preferred to get access of SRH information from schools 512(76.0%) followed by Television 475(70.5%) and Radio 425(63.2%) (Figure 2).

**Factors associated with parent-adolescent communications on SRH issues:** In the bivariate analysis educational status of mothers, adolescents who had positive perception on the importance of discussion on SRH issues with their parents, adolescents whoever got SRH information, adolescents whoever had sexual intercourse, openness of parents to discuss on SRH issues were significantly associated with communication on SRH issues (Table 6). The result of multivariate logistic regression model revealed that mothers who could read and write and had diploma certificate, adolescents who had positive perception on the importance of discussion on SRH issues, adolescents whoever had sexual intercourse and ever got SRH information were variables significantly associated with communication on SRH issues with parents (Table 6). Adolescents, whose mothers could read and write were 2 times more likely to communicate on SRH issues with their parents than those students whose mothers could not read and write (AOR = 2.0; 95% CI: 1.3-3.1); similarly, Students whose mothers had diploma certificate were 2 times more likely to communicate on SRH issues with their parents than those adolescents whose mothers were illiterate (AOR = 2.0; 95% CI: 1.4-2.9). Adolescents, who had positive perception on the importance of discussion on SRH issues with their parents were 2.5 times more likely to discuss on SRH issues than those who did not agree the importance (AOR = 2.5, 95% CI: 1.3-4.5). Those students whoever got SRH information were 2 times more likely to communicate on SRH issues with their parents than those who never got SRH information (OR = 2.0; 95% CI: 1.4-2.9). This study also revealed that those adolescents whoever had sexual intercourse were 1.7 times more likely to communicate on SRH issues with their parents than those who never had sexual intercourse (AOR = 1.7; 95% CI: 1.1-2.6) (Table 6).

**Table 6:** Bivariate and multivariate analysis of factors associated with communication on SRH issues with parents among High school students in Woldia town, December 2016

Variables		Communication on SRH issues with parents	
		Crude OR (95% CI)	Adjusted OR (95% CI)
Sex	Male	0.80(0.37,1.7)	0.78(0.30,2.1)
	Female	0.80(0.37,1.7)	1
Age	15-19	0.84(0.38,1.8)	1
	20-24	1	1.08(0.31,3.7)
Grade level	9	1	1
	10	0.8(0.5, 1.2)	0.7(0.5, 1.1)
Living arrangement	Both parents	1	1
	Single parent	1.96(0.60,6.40)	1.61(0.37,6.9)
	Others	3.08(0.70,13.4)	2.23(0.42,11.7)
Family size	< 5	0.66(0.25,1.7)	1.51(0.41,5.5)
	5 & above		1
Mother’s education status	Illiterate	1	1
	Read & write only	2.2(1.4, 3.3)**	2(1.3, 3.1)**
	Primary school	1.3(0.7, 2.3)	1.1(0.6, 1.9)
	Secondary school	1.7(1.0, 2.8)**	1.4(0.8, 2.3)
	Diploma	2.2(1.6,3.1)**	2(1.4, 2.9)**
	Degree & above	1.6(1.0, 2.7)**	1.3(0.8, 2.2)
Father’s education status	Illiterate	1	1
	Read & write only	0.9(0.5, 1.6)	0.8(0.4, 1.4)
	Primary school	1.7(0.9, 3.1)	1.4(0.7, 2.9)
	Secondary school	1.3(0.7, 2.4)	1.0(0.4, 2.2)
	Diploma	1.1(0.6, 1.8)	0.7(0.3, 1.6)
	Degree & above	0.41[0.18,1.34)	0.23(0.06,1.8)
Occupation of Father	Unemployed	0.8(0.4, 1.6)	0.9(0.4, 2.1)
	Gov’t Employee	0.7(0.3, 1.5)	0.8(0.3, 1.8)
	Private employee	1.1(0.5, 2.1)	1.1(0.5, 2.5)
	Merchant	1.0(0.5, 2.0)	1.0(0.4, 2.2)
	Others	1	1
Occupation of Mother	Housewife	1	1
	Gov’t Employee	1.1(0.5, 2.1)	1.2(0.6, 2.5)
	Private employee	1.3(0.8, 2.1)	1.4(0.7, 2.8)
	Merchant	0.8(0.5, 1.4)	0.7(0.4, 1.4)
	Others	1.3(0.4, 3.7)	1.3(0.4, 4.1)
Knowledgeable about SRH issues	Yes	0.9(0.7, 1.3)	1.0(0.7, 1.5)
	No	1	1
Importance to discuss SRH issues with parent	Yes	2.7(1.5,4.8)**	2.5(1.3, 4.5)**
	No	1	1
Had ever got SRH information	Yes	2.2(1.6,3.1)**	2(1.4, 2.9)**
	No	1	1
Ever had sexual intercourse	Yes	1.5(1.0, 2.2)**	1.7(1.1, 2.6)**
	No	1	1
Openness of Parents	Yes	6.08(3.95, 9.3)*	3.45(2.35, 6.6)*
	No	1	1

** =P=0.001, = P=0.05, 1= constant

## Discussion

In this study, 30.4% of adolescents had discussed on SRH issues with their parents in the last 12 months, which indicates very limited communication. The main reasons for the very limited communication could be conservative norms around sexuality, limited parental SRH knowledge, and fear that such communication would encourage sexual activities. In agreement with this finding the study conducted in Hawassa, Ethiopia reported that 30.4% of adolescents had discussed on SRH issues with their parents [15]. However, our figure is higher than a study conducted in Bullen, Ethiopia (29%)[13], Lesotho (20%) [10], Awabel, Ethiopia (25.3%) [6], but, lower than the study conducted in Dire Dawa, Ethiopia (37%) [16], Debre Markos, Ethiopia (36.9%) [17], Yirgalem, Ethiopia (59.1%) [18] and Zimbabwe (44%) [19]. However, a study conducted in Ghana revealed that 82.3% of parents had at some point in time discussion on SRH issues with their children; nonetheless, the discussions centered on a few topics [20]. This may be due to demographic and cultural difference and the difference in accessing SRH information and youth-friendly health service to address adolescents' SRH desires in Ghana. Thus far, in many of the existing studies, the studies have examined the SRH communication with “yes” or “no” responses, which tell the presence or absence of communication than how frequent the communication occurs. In our study, 227(33.7%) of adolescents had a discussion on STIs/HIV/AIDS. Among those adolescents, 188(27.9%) had a discussion with their friends, followed by their mothers which account 140(20.8%). Two hundred nine (31.1%) of the respondents reasoned out that their mothers were open to discuss SRH issues as compared to their fathers 16.8%. Similarly, a report from Ghana revealed that 78.8% of mothers had discussed sexual communication with their children [20], however, the majority of adolescents preferred to discuss with their peers than their parents [20]. This is consistent with the report by Ayehu and his co-authors, 431(57.8 %) of adolescents had SRH discussion with their peers than parents [6]. This is due to the fact that peers may become more powerful sexual socialization agents than parents, particularly for information about sexual intercourse [5]. In contrast, previous literatures [1, 18, 19] reported that more communications were made with mothers as compared to fathers and peers. In this study, majority of the adolescents (66.3%) had no discussion about STIs/HIV/AIDS with their parents. The adolescents' estimated reason for not discussing about STIs/HIV/AIDS with their parents were parents had less communication skill 199(29.5%), parents lack of knowledge 132(19.6%) and felt ashamed 112(16.6%). Similarly, Tesso and his co-authors [1] reported that fear of parents, cultural taboos, embarrassments and parents' lack of knowledge related to SRH issues were found to be barriers for parent-communications with their children.

Majority of adolescents, (66.1%) in this study mentioned that they got SRH information. Among mentioned sources of information for SRH issues, Television and schools account 56.3% and 49.2%, respectively. This may be due to the fact that currently adolescents are more exposed to Television and information communicated through school mini-media, teachers and the incorporation of SRH matters in their grade level science subjects. In this study, from the assessed factors associated with communication on SRH issues, only mothers who could read and write, adolescents' positive perception on the importance of discussion on SRH issues, adolescents whoever had sexual intercourse and whoever got SRH information showed significant positive association with communication on SRH issues. This result is inconsistent with the study conducted in Debre Markos, Ethiopia [17]. Those adolescents whose mothers could read and write were more likely to communicate SRH issues with their parents than those students whose mothers were illiterate. This is in line with the study conducted from Hawasa, Ethiopia [15] and in Debre Markos, Ethiopia [17]. This may be due to the difference in mothers' knowledge on SRH issues, communication skills, perceived the importance of discussion on SRH issues, and access of information about sexual and reproductive health issues. Adolescents who believed on the importance of discussing SRH matters with their parents were more likely to discuss SRH issues than those who had not believe the importance. This may be due to students' perception difference on the importance of discussion on different SRH issues with their parents. A similar result had reported from Debre-Markos, Ethiopia [17]. Those students whoever got SRH information were more likely to communicate SRH issues with their parents than those who never got SRH information. This may be due to that adolescents who had SRH information would be more aware and keen to discuss SRH issues and the information they got may gave the way for initiation of communication. The study revealed that those adolescents whoever had sexual intercourse were more likely to communicate on SRH issues with their parents than those who never had sexual intercourse. This may be due to fear of risks come as a result of sex and exploration habits of the adolescents.

## Conclusion

In this study parent-adolescent communication on SRH issues was inadequate. Adolescents whose mothers could read and write and adolescents whoever got SRH information had communication on SRH issues with their parents than others. Moreover, adolescents who had positive perception on the importance of discussion on SRH issues with parents and adolescents whoever had sexual intercourse were more likely to communicate SRH issues with their parents as compared to others. Majority of adolescents preferred their friends/peers other than parents to discuss on different SRH issues. School-based education is important to improve adolescent-parent communication about SRH issues. Equip and educate parents on different SRH issues, initiate comprehensive family life education for the adolescents and parents are needed. Moreover, a qualitative study could be done on adolescents and parents communication.

### What is known about this topic

- Adolescent sexual and reproductive health has been overlooked historically despite the high risks that countries face for its neglect; Some of the challenges faced by adolescents across the world include early pregnancy and parenthood, unintended pregnancy, difficulties accessing contraception and safe abortion;
- Young people are currently the group most severely impacted by HIV/AIDS and other STIs;
- Most youth-friendly services and health care providers in Ethiopia are not well prepared in addressing adolescents' SRH desires; healthcare workers often act as a barrier to care by failing to provide young people with supportive, nonjudgmental, youth-appropriate services.

### What this study adds

- Majority of adolescents preferred their peer than parents to discuss all SRH issues; so incorporating peer to peer sexuality education program into school curriculum is needed;
- Adolescents whoever got SRH information were more likely to discuss on some SRH issues with their parents; hence, providing behavioral change communication, conducting sustainable advocacy works targeting parents and communities on young people's SRH is important to improve adolescent-parent communication on SRH issues;
- Programmes/policies related to youth and adolescents' SRH should address not only individual or behavioral factors but also cultural and social factors that negatively influence parents' communication on SRH issues.

## Competing interests

The authors declare no competing interests.
